# The Portevin–Le Chatelier Effect of Cu–2.0Be Alloy during Hot Compression

**DOI:** 10.3390/ma16124455

**Published:** 2023-06-18

**Authors:** Daibo Zhu, Na Wu, Yang Liu, Xiaojin Liu, Chaohua Jiang, Yanbin Jiang, Hongyun Zhao, Shuhui Cui, Guilan Xie

**Affiliations:** 1 School of Mechanical Engineering and Mechanics, Xiangtan University, Xiangtan 411105, China; daibozhu@xtu.edu.cn (D.Z.); m17873820325@163.com (N.W.); liuxiaojin11225@126.com (X.L.); 202005501512@smail.xtu.edu.cn (C.J.); 2 School of Materials Science and Engineering, Nanyang Technological University, Singapore 639798, Singapore; 3 School of Materials Science and Engineering, Central South University, Changsha 410083, China; jybin8113@163.com; 4 State Key Laboratory of Special Rare Metal Materials, Northwest Rare Metal Materials Research Institute Ningxia Co., Ltd., Shizuishan 753000, China; yunhongzhao2023@163.com (H.Z.); shuhuicui2023@163.com (S.C.)

**Keywords:** Cu–Be alloy, Portevin–Le Chaterlier effect, serrations, dynamic phase transformation

## Abstract

The Portevin–Le Chatelier effect of Cu–2.0Be alloy was investigated using hot isothermal compression at varying strain rates (0.01–10 s^−1^) and temperature (903–1063 K). An Arrhenius-type constitutive equation was developed, and the average activation was determined. Both strain-rate-sensitive and temperature-sensitive serrations were identified. The stress–strain curve exhibited three types of serrations: type A at high strain rates, type B (mixed A + B) at medium strain rates, and type C at low strain rates. The serration mechanism is mainly affected by the interaction between the velocity of solute atom diffusion and movable dislocations. As the strain rate increases, the dislocations outpace the diffusion speed of the solute atoms, limiting their ability to effectively pin the dislocations, resulting in lower dislocation density and serration amplitude. Moreover, the dynamic phase transformation triggers the formation of nanoscale dispersive *β* phases, which impede dislocation and cause a rapid increase in the effective stress required for unpinning, leading to the formation of mixed A + B serrations at 1 s^−1^.

## 1. Introduction

The Portevin–Le Chatelier (PLC) effect, a well-known phenomenon first observed by Portevin and Le Chatelier [1,2], occurs in a large number of industrial alloys such as aluminum alloys, steel, and copper alloys [3,4,5]. During the plastic deformation process, the flow stress, work hardening, and ductility can be influenced by the PLC effect, which manifests as serrations on the stress–strain curve [5,6]. The extrinsic factors of the PLC effect are mainly the deformation temperature and strain rate, whereas the intrinsic causes include the size and concentration of atoms, solute diffusivity, precipitation characteristics, and dislocation patterns. Dynamic strain aging (DSA), which is defined as the interplay between movable dislocations and the diffusion of solute atoms, is the primary mechanism underlying the PLC effect [7,8,9]. DSA produces a variety of types of heterogeneous deformation, which are divided into three main stress–strain curve serration-types [6,10]:

- The type A serration is usually found at high strain rates and low deformation temperatures, exhibiting relatively small fluctuations in flow stress, and the fluctuations appear at a random frequency in the stress–strain curve. The main reason for this behavior is the locking of movable dislocations by the solute atoms.
- The type B serration, which usually occurs at medium to high strain rates, is characterized by rapid continuous oscillations above and below the average stress value, obtaining higher amplitudes than those of type A serrations. Under certain conditions, the type B serration could exhibit a minor drop in stress within a certain interval.
- The type C serration is commonly found at relatively low strain rates and exhibits high amplitude and frequency stress oscillations. Type C serration oscillations are above and below the mean value, with larger amplitudes than those of type B serrations.

The PLC effect in copper alloys has been studied in Cu–Al [11], Cu–Cr–Nb [12], Cu–Ga [13], Cu–Sn [14], Cu–Si [15], Cu–Ti [15], Cu–P [15], Cu–Zn [16], and Cu–Ni–Zn [17] copper alloy systems. For Cu–Be alloys [18,19], researchers have focused on the serrations under various aging temperatures (593 K—733 K) and strain rates, due to the precipitation process, which is well-known as G.P. zones—*γ′′* ordered phase—*γ′* phase—equilibrium *γ* phase [20,21,22]. Previous work on the PLC effects is beneficial for the shape forming of Cu–Be sheets. However, the wide application of Cu–Be alloys in industrial applications is not limited to manufacturing various geometric and near-net shapes, It, also includes the production of sheet/foil/bar/wire materials [23,24], the processing temperature of which is higher than 893 K. At the deformation temperatures above 893 K, the *β* phases are precipitated through dynamic phase transformation, the diffusion rate of which is higher than that of the traditional aging precipitations, and the effect of *β* phases on serrations of the Cu–Be alloy is not clear at present. Hence, comprehensively understanding the serrated flow behavior and serration mechanism of Cu–Be alloys at high deformation temperatures is of great significance. To investigate the PLC effect and serration types of Cu–2.0Be alloy at various deformation conditions, hot isothermal compression deformation was employed in this work. The Arrhenius model was chosen to calculate the thermal activation energy and decouple the constitutive relationship under various deformation conditions. In addition, the serration mechanism under various deformation conditions was illustrated.

## 2. Experimental Process

**Figure 1:**
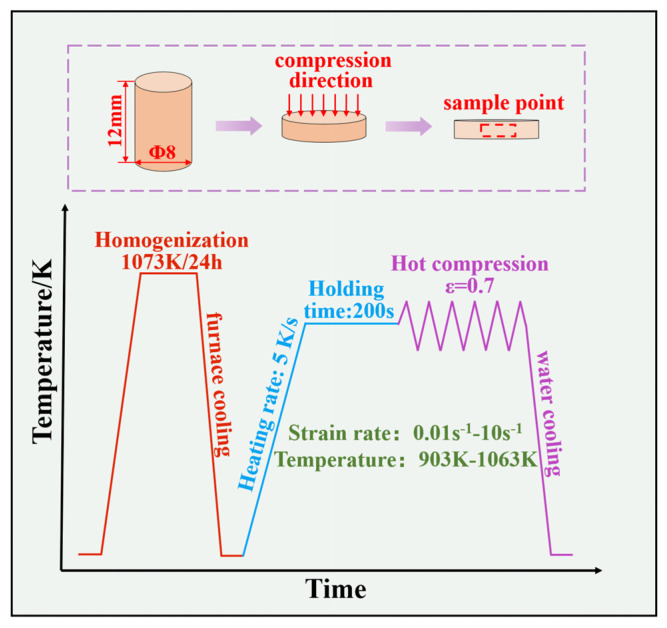
Schematic diagram of hot compression experiment process.

Table 1 lists the component (in wt.%) of the Cu–Be alloy used in this study. A homogenization heat treatment at 1073 K for 24 h was used to prepare all the samples, and then cooled with the furnace for 36 h. For hot compression testing, cylindrical samples with a diameter of 8 mm and height of 12 mm were selected using a Gleeble-3500 thermomechanical simulator with a heating rate of 5 K/s. To achieve consistent heat deformation, all samples were maintained at the necessary temperature for 5 min. In order to reduce the effect of friction between the sample and the processing platform, graphite lubricant with a thickness of approximately 1 mm was applied to coat on the flat end of the sample. The experimental parameters were as follows: for a true strain of 0.7, the deformation temperatures ranged from 903 to 1063 K with a 40 K interval, whereas the strain rates were 0.01 s^−1^, 0.1 s^−1^, 1 s^−1^, and 10 s^−1^. All compression tests were carried out in a vacuum, and all samples were promptly water-quenched to maintain the deformed microstructure following the hot compression tests. The samples for microstructural analysis were cut along the longitudinal plane following the hot compression tests. Figure 1 depicts the hot compression experimentation process.

Samples for electron backscatter diffraction pattern (EBSD) analysis were mechanically and vibratory polished for 0.5 h by a Buehler VibroMet2 Vibratory Polisher. A field emission gun-equipped FEl scanning electron microscope was used to characterize the grain structure. The HKL Channel 5 software (VMware Workstation 15.5.5 Pro) was chosen to analyze the EBSD data using a step size of 0.6 um. Low-angle grain boundaries (LAGBs) with orientations between 2° and 10° in the EBSD maps are depicted by red lines, whereas the high-angle grain boundaries (HAGBs) with orientations >10° are depicted by blue lines. Transmission electron microscopy (TEM) images were collected using a JEM-2100F transmission electron microscope at 200 kV. The TEM sample was formed into a disc with a 50 μm thickness and 3 mm diameter, and twin-jet thinning was applied in a mixed solution of HNO_3_:CH_3_OH = 1:3. The experimental voltage and temperature were 30 V and –30 °C respectively.

## 3. Result and Discussion

### 3.1. The Influence of the Temperature and Strain Rate on the Serrated Flow

**Figure 2:**
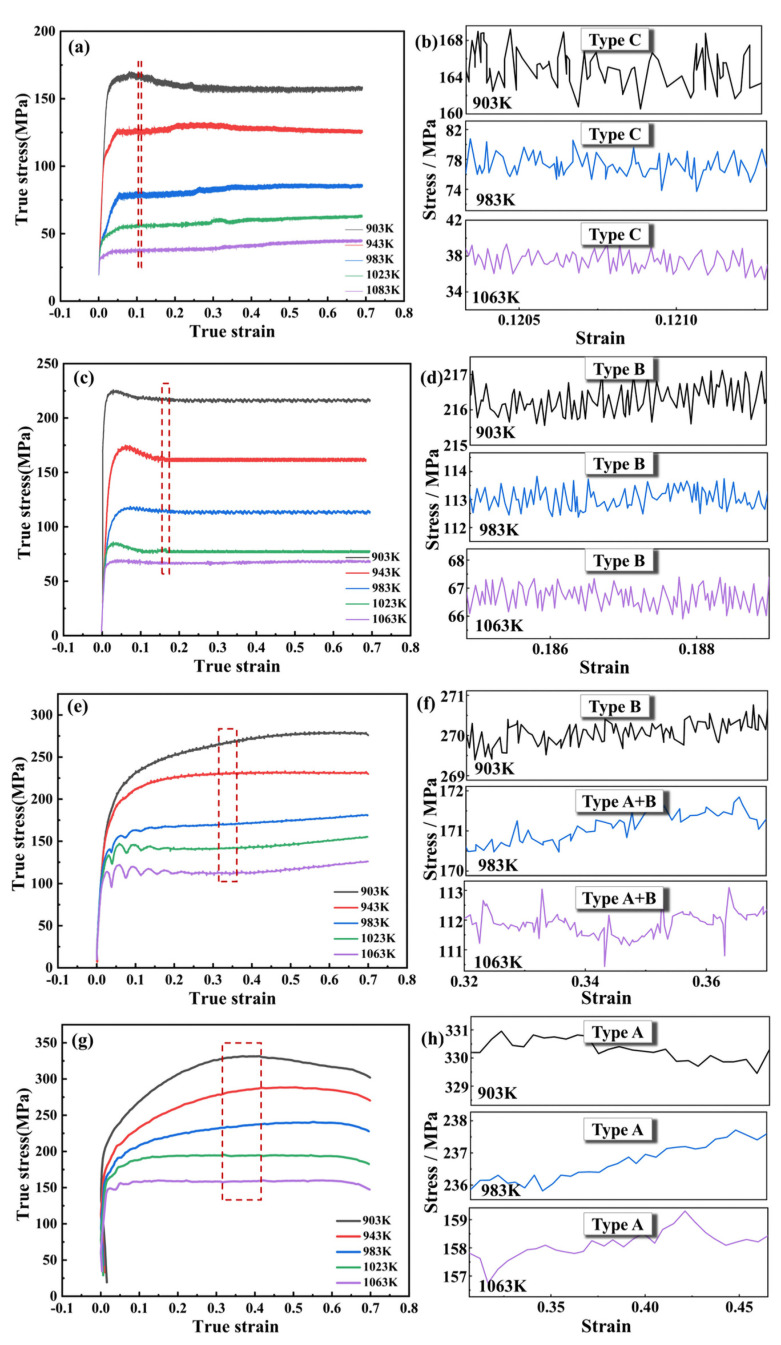
Flow stress curves of the Cu–2.0Be alloy at various strain rates and temperatures: (**a**) 0.01 s^−1^, (**c**) 0.1 s^−1^, (**e**) 1 s^−1^, and (**g**) 10 s^−1^. (**a**,**c**,**e**,**g**) are magnified views of the red dashed boxes, respectively, in (**b**,**d**,**f**,**h**).

The true flow stress curves of hot isothermal deformation are shown in Figure 2 for strain rates of 0.01 s^−1^ (Figure 2a), 0.1 s^−1^ (Figure 2c), 1 s^−1^ (Figure 2e), and 10 s^−1^ (Figure 2g), with magnified views of the red dashed box given in Figure 2b,d,f,h, respectively. According to the results, the compression temperature and strain rate were closely related to the flow stress change. At a certain strain rate, the flow stress decreased with rising temperature. Additionally, the flow stress rose with an increasing strain rate at a certain deformation temperature. The development of the flow stress curves can be summarized as occurring in three stages. In the first stage, the flow stress increased practically linearly with increasing strain (the occurrence of work hardening), which was caused by the development and propagation of dislocations. In the second stage, the increasing rate of flow stress slowed down and reached its peak value, indicating the emergence of dynamic restoration (flow softening) processes, such as dynamic recovery (DRV) and dynamic recrystallization (DRX). In the third stage, DRX appeared, and the flow stress decreased and eventually began to level off.

Based on the magnified views of flow stress in Figure 2, with increasing strain rate, the serration changed from type C (Figure 2a) to type B (Figure 2c,e) and eventually to type A (Figure 2g). Table 2 displays the distribution of serration types in accordance with the temperature and strain rate. The decisive factor affecting the serration was obviously the strain rate. Furthermore, the temperature also affected the serration type under a certain strain rate (1 s^−1^ in Figure 2e). When the compression temperature was above than 943 K, the type of serration changed from type B to type A + B. The serration transformation rules of the Cu–2.0Be alloy are not affected by the deformation temperature in the same manner as other copper alloy works with regard to hot compression [4,25,26,27,28,29].

**Figure 3:**
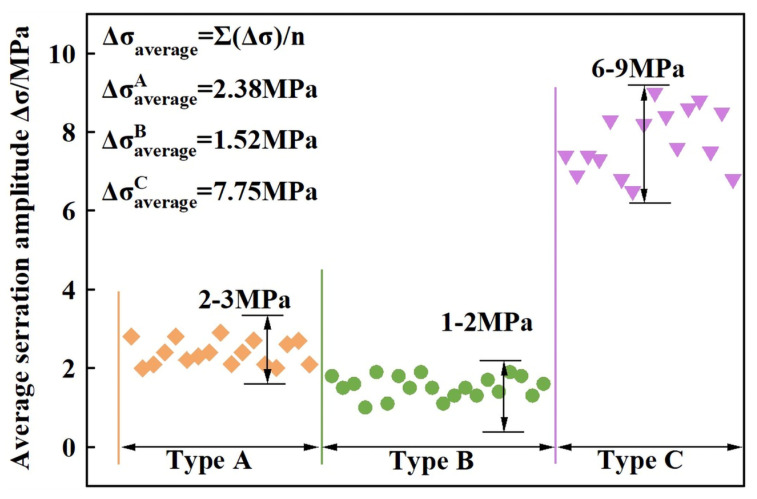
Statistics of variation in the average serration amplitude of various serration types. Serrations of types A, B, and C have average amplitudes of 2~3 MPa, 1~2 MPa, and 6~9 MPa, respectively.

The amplitudes of the serrations represent the degree to which the stress deviates away from the average level. The average serration amplitudes of the various serration types at the corresponding temperatures are shown in Figure 3. The type C serration had the greatest amplitude (7.75 MPa), and the ordinary serration amplitudes of type A and type B (type A + B) serrations were approximately 2.38 MPa and 1.52 MPa, respectively. Low strain rate causes high concentration of clustered solutes at dislocations during the effective waiting time, resulting in type C serrations. Therefore, a high average strength will be needed for unpinning, and larger amplitudes could be obtained compared with those of type A or B serrations [30].

### 3.2. Constitutive Equation and Activation Energy

In order to obtain the activation energy under different strain rates, the Arrhenius equation was employed. The plastic flow behavior during the deformation processes was described using constitutive equations connected to different deformation situations. Under both low- and high-stress circumstances, the hyperbolic sine function of the Arrhenius equation may typically be reduced to an exponential and power function. Equations (1) and (2) illustrate how the deformation circumstances affect the thermal working behavior of the Cu–2.0Be alloy using the Arrhenius-type constitutive model [31].

(1) $$Z=A[\sinh(a\sigma ){]}^{S}=\dot{\varepsilon }\exp(Q/RT)$$

(2) $$\dot{\varepsilon }=\{\begin{array}{c} A{\left[\sinh\left(a\sigma \right)\right]}^{n}\exp\left(-\frac{Q}{\mathrm{RT}}\right),\;for\;all\;values\;of\;a\sigma \;\\ A_{1}\sigma^{n_{1}}\exp\left(-\frac{Q}{\mathrm{RT}}\right),\;if\;a\sigma <0.8\\ A_{2}\exp\left(\beta \sigma \right)\exp\left(-\frac{Q}{\mathrm{RT}}\right),\;if\;a\sigma >1.2 \end{array}$$

where A, A_1_, A_2_, n, n_1_, *a* and *β* are material constants, $\dot{\varepsilon }$ is the strain rate (s^−l^), R is the gas constant, *σ* is the flow stress (MPa), T is the absolute temperature (K), and Q is the thermal activation energy needed during plastic deformation. The *α* can be defined as $a=\beta /n_{1}$.

The logarithm of the above equation results in [32,33]:

(3) $$\ln\dot{\varepsilon }=\{\begin{array}{c} \mathrm{lnA}-\frac{Q}{\mathrm{RT}}+\mathrm{nln}\left[\sinh\left(\sigma \right)\right]\\ \ln A_{1}-\frac{Q}{\mathrm{RT}}+n_{1}\ln\left(\sigma \right)\\ \ln A_{2}-\frac{Q}{\mathrm{RT}}+\beta \sigma \end{array}$$

**Figure 4:**
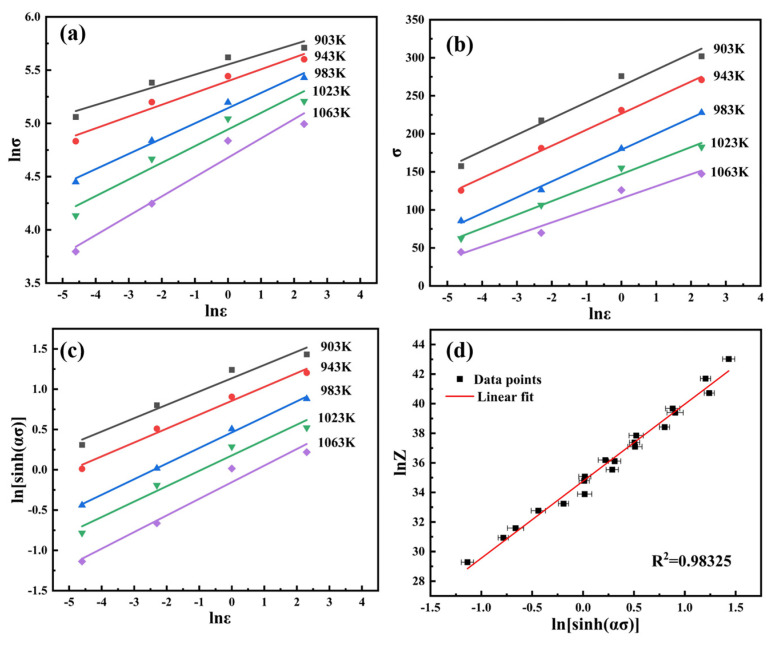
The flow stress and strain rate relationship: (**a**) lnσ~$\ln\dot{\varepsilon }$, (**b**) σ~$\ln\dot{\varepsilon }$, (**c**) ln[sinh(aσ)]~$\ln\dot{\varepsilon }$, (**d**) lnZ~ln[sinh(aσ)].

Thus, through analyzing the slope of the curve in the Figure 4a–c, the value of 1/n = 0.19455, 1/n_1_ = 0.148258, and 1/*β* = 15.00435 can be obtained, respectively. The value of ln*A* = 34.76345 can be calculated by taking the logarithm of Equation (1) and fitting it to the intercept of the line in Figure 4d.

(4) lnZ= lnA+sln[sinh(aσ)]

**Figure 5:**
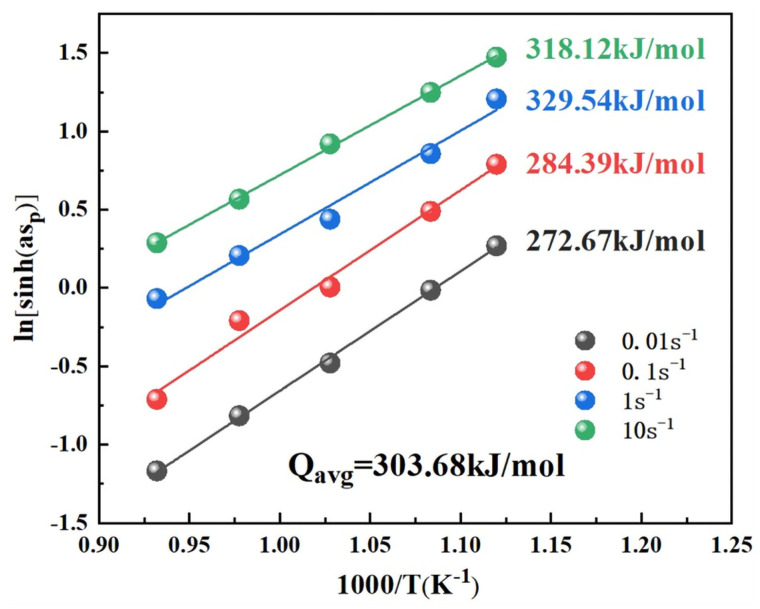
Calculation of the activation energy for serrated flow Plot of ln[sinh(aσ)] versus 1/T.

The value of Q can be obtained by linear fitting in Figure 5, which can be expressed by deducing Equation (3):

(5) $$Q\;=\;\mathrm{Rs}\frac{d\{\ln\left[\sinh\left(\alpha \sigma \right)\right]\}}{d\left(1/T\right)}$$

The activation energy decreases with strain rate enhancement, and the activation energy (Q) is 303.68 kJ/mol on average. According to the above values, the constitutive equation of hot compression can be expressed as follows:

(6) $$\dot{\varepsilon }=e^{34.76}{\left[\sin h\left(0.0071\sigma \right)\right]}^{5.14}\exp\left(-\frac{303.68}{\mathrm{RT}}\right)$$

### 3.3. Microstructure Evolution

**Figure 6:**
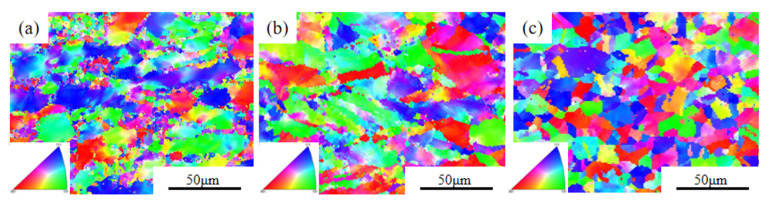
EBSD micrographs of the Cu–2.0Be alloy deformed at: (**a**) 903 K, 1 s^−1^; (**b**) 983 K, 1 s^−1^; (**c**) 1063 K, 1 s^−1^.

The EBSD diagrams of the deformed Cu–2.0Be alloy samples are shown in Figure 6. When the deformation conditions were 903 K/1 s^−1^, large grains dominated, as shown in Figure 6a. A small amount of recrystallization occurred at the grain boundaries of the large grains, and the original grains of the alloy elongated. When the temperature was increased from 903 to 1063 K, dynamic recrystallization occurred, the region became larger, and the grain growth direction changed from an “item chain” distribution to a spherical distribution, as shown in Figure 6c.

**Figure 7:**
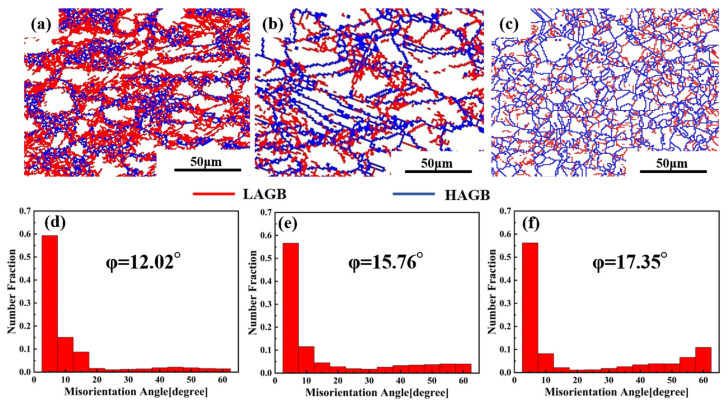
HAGBs and LAGBs distribution maps and misorientation angle distributions of the Cu–2.0Be alloy deformed at: (**a**,**d**) 903 K, 1 s^−1^; (**b**,**e**) 983 K, 1 s^−1^; (**c**,**f**) 1063 K, 1 s^−1^. LAGBs are depicted as red lines, while HAGBs are displayed as blue lines.

**Figure 8:**
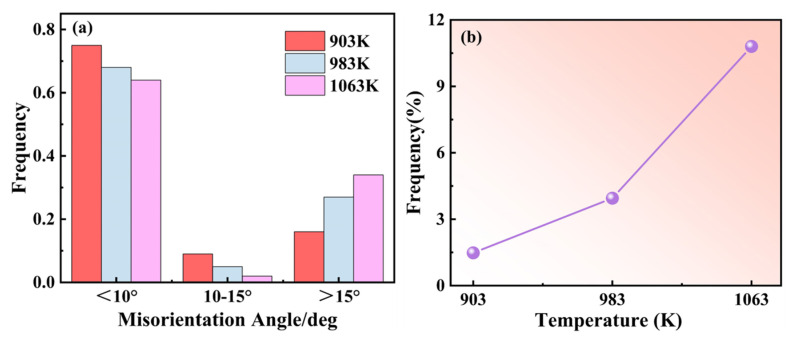
Diagram of grain boundary characteristics: (**a**) misorientation angle scope variations; (**b**) the distribution of TBs.

**Figure 9:**
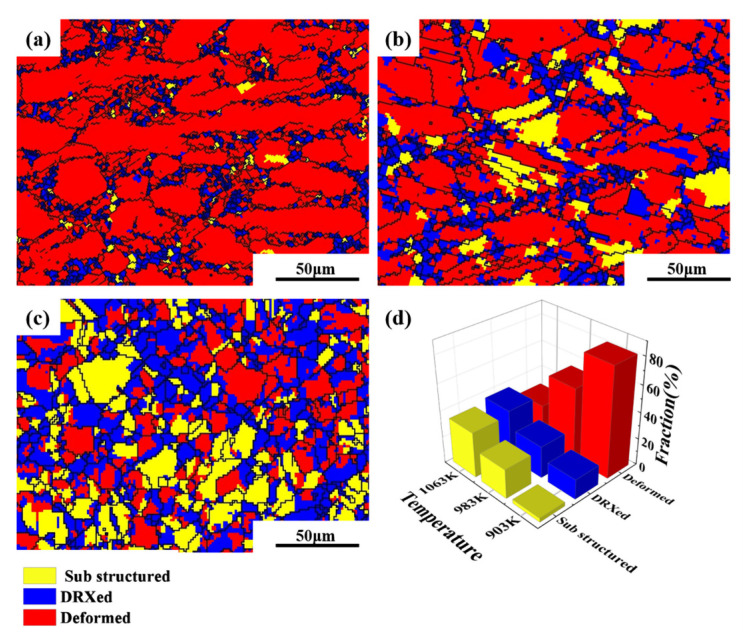
Recrystallization grain distribution of the Cu–2.0Be alloy deformed at: (**a**) 903 K, 1 s^−1^; (**b**) 983 K, 1 s^−1^; (**c**) 1063 K, 1 s^−1^; (**d**) distribution statistics chart.

**Figure 10:**
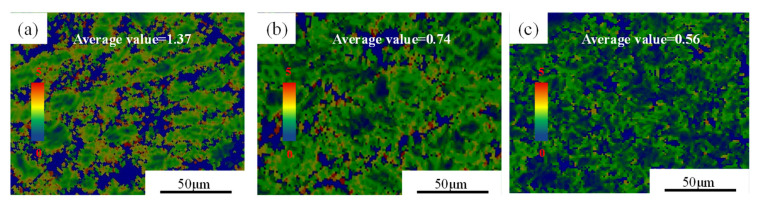
The kernel average misorientation (KAM) of the Cu–2.0Be alloy deformed at: (**a**) 903 K, 1 s^−1^; (**b**) 983 K, 1 s^−1^; (**c**) 1063 K, 1 s^−1^.

The LAGBs and HAGBs distribution maps and misorientation angle distributions of the Cu–2.0Be alloy samples are shown in Figure 7. Due to the accumulation of dislocations in the deformed grains, many LAGBs formed at 903 K, as seen in Figure 7a. The development of recrystallized grains caused LAGBs to become HAGBs as the deformation temperature rose. Additionally, in Figure 7d,f, the average misorientation angle value rose from 12.02 to 17.35. Based on the statistical data in Figure 8a, the volume percentage of HAGBs increased from 16.4 to 35.4% with increasing temperature, indicating that the degree of recrystallization was further enhanced with increasing HAGBs [34]. Particularly, the fraction of HAGBs close to 60° has increased can be seen in Figure 7d–f, which indicates the presence of annealing twin boundaries (TBs). Further, the distribution of the percentage of TBs is shown in Figure 8b. It can be found that the percentage of TBs increased from 1.5% to 11% with the temperature increase. The recrystallized grain distribution of the hot compressed samples is shown in Figure 9. At 903 K (Figure 9a), the DRX is suppressed due to the low deformation temperature, and there are more red inhomogeneous deformed tissues. With the temperature enhancement, the deformed grains gradually transform to the recrystallized grains through the nucleation and growth of new grains, which results in the increase the proportion of the blue DRXed grains and yellow Sub-structured grains. The kernel average misorientation (KAM) maps in Figure 10 can be used to determine the dislocation density variations and distributions. The average dislocation density value fell as the deformation temperature rose, indicating the presence and coarsening of DRX sacrifice dislocations [35]. Furthermore, the region of high dislocation density is primarily localized close to the grain boundaries shown in Figure 10. The dislocation density was substantially higher in the grain boundary area than within the grains, indicating the preferential occurrence of DRX in the grain boundary area.

**Figure 11:**
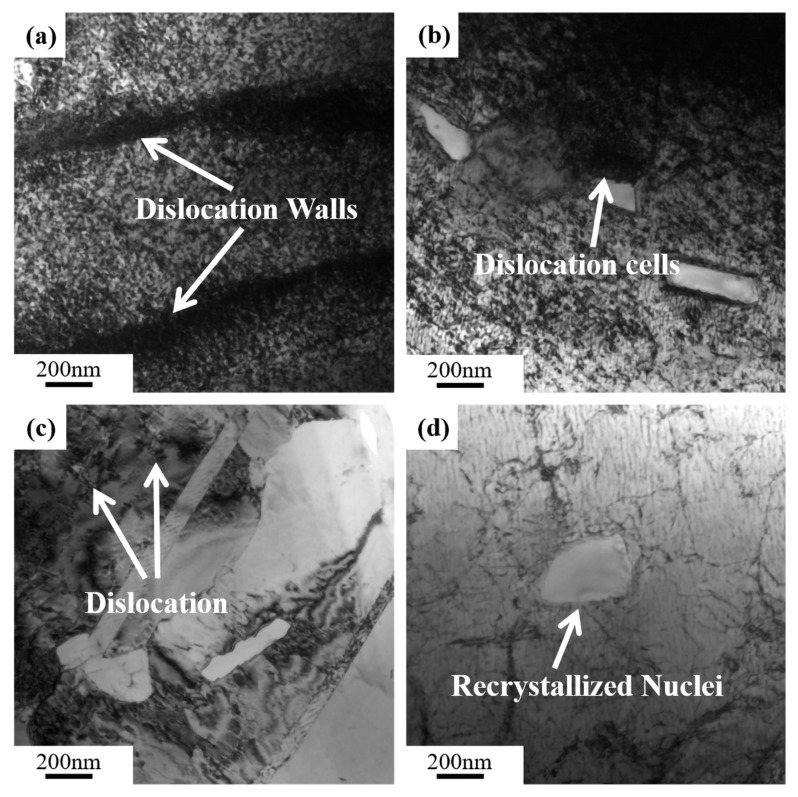
TEM images along with the compressive temperature of 903 K at various strain rate: (**a**) 0.01 s^−1^; (**b**) 0.1 s^−1^; (**c**) 1 s^−1^; (**d**) 10 s^−1^.

**Figure 12:**
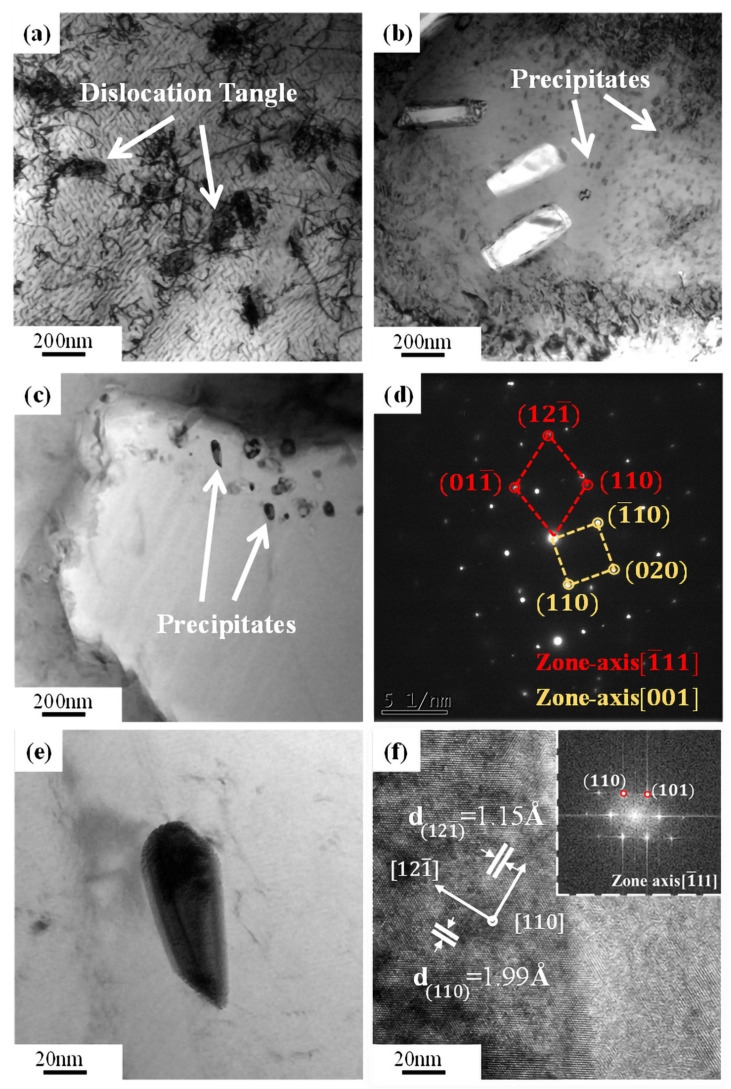
TEM images along with the compressive strain rate of 1 s^−1^ at different temperature: (**a**) 943 K; (**b**) 983 K; (**c**) 1063 K, (**d**) represents the selected area electron diffraction (SAED) patterns of (**c**), (**e**) is a magnified view of (**c**), and (**f**) is the HRTEM of (**e**).

To clarify the effect of the compression rate and temperature on the serration types, compressed samples under different conditions were analyzed by TEM/HRTEM. The results are shown in Figure 11 and Figure 12.

Figure 11 is images of samples with different strain rates at 903 K. The dislocations were gradually unlocked as the strain rate increased, leading to the emergence of the different serration mechanisms shown in Figure 2. When the strain rate was relatively low in Figure 11a, the dislocation movement velocity was lower than the diffusion velocity of solute atoms. Hence, the dislocations were effectively pinned, resulting in a higher amplitude and frequency of serrations in the flow stress curves (type C serrations) [36]. As strain rates rose, the dislocation movement velocity gradually outpaced the rate at which solute atoms diffused, leading to the unlocking of dislocations. At medium strain rates, type B serrated flow was achieved [37]. When the strain rate was 10 s^−1^, the dislocation movement speed was faster than the rate of solute atom diffusion, and a lower dislocation density was obtained Figure 11d, generating the minor, random undulation of type A serrations in the stress–strain curves [30]. Moreover, with the gradual unlocking of dislocations, the typical dislocation-induced recrystallization can be seen in Figure 11b–d.

TEM images of hot compressed samples at a strain rate of 1 s^−1^ and temperatures of 943 K, 983 K, and 1063 K are shown in Figure 12. At 943 K, the precipitated phases had a small size and a large number of dislocation tangles (Figure 12a). The size of the precipitated phases steadily grew from 5–15 nm (Figure 12b) to 80–160 nm (Figure 12c) as the temperature rose, while the density of dislocations gradually decreased. Furthermore, Figure 12d shows the SAED of the precipitate in Figure 12c, demonstrating a body center cubic (BCC) structure. Figure 12e displays the block-shaped precipitate’s high-resolution TEM (HRETEM) micrograph and accompanying Fast Fourier Transform (FFT) pattern in Figure 12f. The distance between the layered precipitates, called *d*-spacing, is around 1.99 Å, which is close to that of the *(hkl) =* (110) plane of the BCC structure found in the diffraction pattern database of the *β* phase [38,39,40].

**Figure 13:**
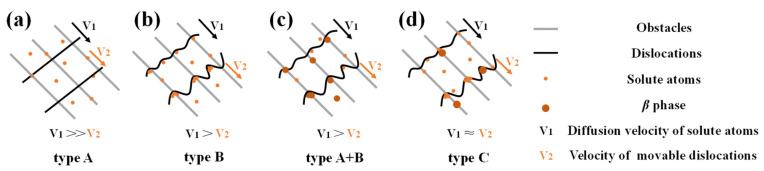
The sketch map of the serration mechanism process in type (**a**) A, (**b**) B, (**c**) A + B and (**d**) C.

The mechanism of serration (Figure 13) is essentially the interaction between the dislocation motion (grain boundary motion) and the diffusion of solute atoms [41]. As illustrated in Figure 13a, when the strain rate is large enough, such as 10 s^−1^, the amounts of solute atoms caught by excessive dislocation movement speed to form entanglement is greatly reduced under the influence of external force. As a result, the dislocation density is relatively low in Figure 11d, only a small amount of serration occurs randomly, and the serration frequency is greatly reduced in Figure 2g,h. Furthermore, insufficient time passes to generate the *β* phase, which results in an extremely small serration amplitude (type A). The same phenomenon is also well verified in the articles on copper alloys [42], nickel-based superalloys [36], magnesium alloys [30] and steels [43]. However, as illustrated in Figure 13b–d, when the strain rate is lower than 10 s^−1^, the reduced strain rate allows enough time for the mobile dislocation to be effectively pinned and generate the *β* phase, which can pin the mobile dislocation ulteriorly. The effective stress needed for unpinning is enhanced, increasing the amplitude of the serration and dislocation density. The lower the strain rate, the higher the required effective stress and the larger the serration amplitude. As a result, the frequency and amplitude of type C serrations are the largest among all the stress–strain curves in Figure 2a.

Normally, despite the fact that the speed of solute atom diffusion increases as the temperature rises, the development of grains by DRX significantly reduces the number of dislocations in the grain border and matrix (Figure 6, Figure 7, Figure 8, Figure 9 and Figure 10), which results in a serration amplitude that is not sensitive to temperature at certain strain rate. Meanwhile, at a particular strain rate, such as 1 s^−1^, when the temperature is higher than 903 K, a mixed type of serration between types B and A emerges, which is characterized by an abruptly large serration in a small serration. This mixed A + B type of serration combines the characteristics of type A, the serration amplitude of which is large, unstable, and random, and type B, the serration amplitude of which moves up and down steadily with a specific number continuously. As shown in Figure 12, nanoscale dispersive *β* phases will be readily formed at high temperatures (983 K and 1063 K) at 1 s^−1^, which can hinder the movement of the dislocations and lead to the rapid increase in the effective stress required for unpinning. As a result, mixed A + B type serrations are generated, as illustrated in Figure 13c.

## 4. Conclusions

To investigate the PLC effect of Cu–2.0Be alloy, the hot compression method was employed at temperatures ranging from 903 to 1063 K with strain rates of 0.01 to 10 s^−1^, and the characteristics of serrated flow were studied. In addition, EBSD and TEM microscopic analyses were performed to explore the mechanisms of different serration types. The main conclusions are as follows:

(1) The PLC effect of Cu–2.0Be alloy is sensitive to the strain rate. Types A, B and C serrations occur at high, medium and low strain rates, respectively. In particular, at a strain rate of 1 s^−1^, temperature-sensitive serrations were discovered, and mixed type A + B serrations were produced when the temperature exceeded 983 K.
(2) The Arrhenius-type constitutive equation was created using the stress–strain data in the following manner: $\dot{\varepsilon }=e^{34.76}{\left[\sin h\left(0.0071\sigma \right)\right]}^{5.14}\exp\left(-\frac{303.68}{\mathrm{RT}}\right)$. The activation energy increased with increasing strain rate and the ordinary activation energy Q = 303.68 kJ/mol.
(3) The serrations type is determined by the interaction between the dislocation motion (grain boundary motion) and the diffusion of solute atoms. The flow stress serration is very sensitive to the pinning and unpinning of the mobile dislocations, which can be mainly affected by the solute atom diffusion velocity.
(4) Under appropriate deformation conditions, the formation of fine *β* phases can hinder dislocation movement, resulting in temperature-sensitive serrations and the formation of the mixed type A + B serrations.

## Figures and Tables

**Table 1:** Components of Cu–2.0Be alloy.

Component	Be	Fe	Al	Si	Pb	Others	Cu
Content (wt.%)	2.02	0.045	0.009	0.02	0.0018	0.23	Bal.

**Table 2:** Summary of serrations types observed on stress–strain data.

$\dot{\varepsilon }\;($ $s^{-1})$	Temperature (K)				
	903	943	983	1023	1063
0.01	C	C	C	C	C
0.1	B	B	B	B	B
1	B	B	A + B	A + B	A + B
10	A	A	A	A	A

## Data Availability

The data are available from the corresponding author upon reasonable request.
